# The MetroPT dataset for predictive maintenance

**DOI:** 10.1038/s41597-022-01877-3

**Published:** 2022-12-13

**Authors:** Bruno Veloso, Rita P. Ribeiro, João Gama, Pedro Mota Pereira

**Affiliations:** 1 University Portucalense, Porto, 4200-072 Portugal; 2 INESC TEC, Porto, 4200-465 Portugal; 3 FEP - University of Porto, Porto, 4200-465 Portugal; 4 FCUP - University of Porto, Porto, 4150-177 Portugal

**Keywords:** Scientific data, Computer science

## Abstract

The paper describes the MetroPT data set, an outcome of a Predictive Maintenance project with an urban metro public transportation service in Porto, Portugal. The data was collected in 2022 to develop machine learning methods for online anomaly detection and failure prediction. Several analog sensor signals (pressure, temperature, current consumption), digital signals (control signals, discrete signals), and GPS information (latitude, longitude, and speed) provide a framework that can be easily used and help the development of new machine learning methods. This dataset contains some interesting characteristics and can be a good benchmark for predictive maintenance models.

## Abstract (summary)

**Table:** 

Measurement(s)	pressure of air • Temperature • ampere
Technology Type(s)	Pressure Sensor Device • Temperature Sensor Device • Electrical Current
Sample Characteristic - Environment	trains
Sample Characteristic - Location	Portuguese Republic

## Background & Summary

The occurrence of faults in public transport vehicles during their regular operation is a source of numerous damages, mainly when they cause the interruption of the trip. The negative impacts affect not only the operator company but the clients, who are thereby disappointed with their expectations of transportation trust. In this context, the early detection of such faults can avoid the cancellation of trips and the withdrawal of service from the respective vehicle and thus is of enormous value. Only in 2017, more than 170 trips were cancelled for this reason.

The Air Production Unit (APU) installed on the roof of Metro vehicles feeds units that perform different functions. One of these units is the secondary suspension, responsible for maintaining the height of the vehicle level regardless of the onboard number of passengers. The APU is a highly demanded element on the vehicle throughout the day. The absence of redundancy causes its failure to result in the immediate removal of the train for repair. The failures are typically undetectable according to traditional condition-based maintenance criteria (predefined thresholds).

From the operational point of view, the objective of Predictive Maintenance is to reduce operational problems, reduce the number of unforeseen stops and the stopping time, and change the maintenance paradigm: from reactive to predictive.

In the last few years, many works have been published about Predictive Maintenance (PdM) with the development of machine and deep learning techniques. Recent publications include a survey in Predictive Maintenance^1^ that covers the main issues in data-driven PdM; another survey^2^ describing advances using machine learning and deep learning techniques for handling PdM in the railway industry; and a manuscript^3^ that identifies three key open research lines for the PdM domain: failure prediction, remaining useful life (RUL), and root cause analyses (RCA).

The final goal of PdM consists in timely predicting developing and unexpected failures based on the continuously monitored condition of the equipment. The maintenance plan is dynamically scheduled to reduce unplanned downtime and associated costs. Additionally, by identifying the components involved and the severity of the failure ultimately yields more effective recovery plans.

The MetroPT dataset is a real-world dataset where the ground truth of anomalies is known from the company’s maintenance reports. The objective is that it can be used as a benchmark dataset for Predictive Maintenance, where It will allow for fair comparisons between Machine Learning algorithms developed to detect anomalies based on sensor data collected as a continuous data flow.

## Methods

A signal acquisition system was installed in the Air Production Unit (APU) of a train. The acquisition system follows a rigorous set of protocols and norms required to be used on railway vehicles:

- EN 45545 - Railway applications - Fire protection on railway vehicles
- EN 50121 - Railway applications - Electromagnetic compatibility
- EN 50125 - Railway applications - Environmental conditions for equipment
- EN 50128 - Railway applications - Communication, signalling and processing systems - Software for railway control and protection systems
- EN 50129 - Railway applications - Communication, signalling and processing systems - Safety-related electronic systems for signalling
- EN 50153 - Rolling stock - Protective provisions relating to electrical hazards
- EN 50155 - Railway applications - Electronic equipment used on rolling stock
- EN 60529 - Specification for degrees of protection provided by enclosures (IP code)
- EN 61373 - Railway applications – Rolling stock equipment – Shock and vibration tests
- IEC 60068 - Environmental testing
- IEC 60571 - Electronic equipment used on rail vehicles
- IEC 61375-1 - Electronic railway equipment – Train communication network (TCN) – Part 1: General architecture
- IEC 61375-2-1 - Electronic railway equipment – Train communication network (TCN) – Part 2-1: Wire Train Bus (WTB)
- IEC 61375-3-1 - Electronic railway equipment – Train communication network (TCN) – Part 3-1: Multifunction Vehicle Bus (MVB)

**Fig. 1:**
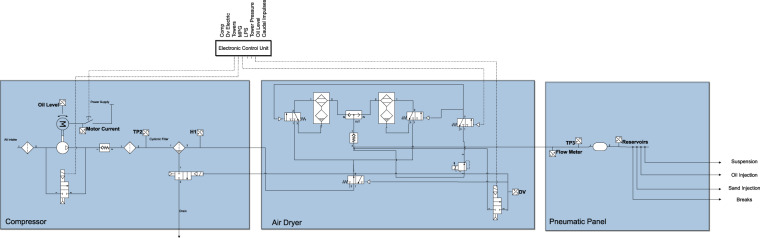
Air Producing Unit (APU).

Figure 1 depicts the components of an APU. The data acquisition rate is 1 Hz, and the information is sent to the remote server every five minutes using the GSM network.

The data collection of the the unit began on 12 March 2020 and is continuously operational to date (presently July 2022). Every day, a report is generated with the information on the sensor signals.

The system installed in the vehicle’s APU collects data from eight analog sensors and eight digital signals. The selection of the sensors was based on an FMEA (Failure Mode and Effects Analysis) and FMECA (Failure Mode, Effects and Criticality Analysis) of the APU. These two analyses were developed by the maintenance teams of Metro of Porto.

### Analog sensors

As for the analog sensors, we have pressure, temperature and electric current consumed at different components of the APU, as detailed below.

- TP2^4^ - Pressure on the compressor (bar).
- TP3^4^ - Pressure generated at the pneumatic panel (bar).
- H1^4^ - Valve that is activated when the pressure read by the pressure switch of the command is above the operating pressure of 10.2 bar (bar).
- DV_pressure^4^ - Pressure exerted due to pressure drop generated when air dryers towers discharge the water. When it is equal to zero, the compressor is working under load (bar).
- Reservoirs^4^ - Pressure inside the air tanks installed on the trains (bar).
- Oil_Temperature^5^ - Temperature of the oil present on the compressor (°C).
- Flowmeter^6^ - Airflow was measured on the pneumatic control panel (m^3^/h).
- Motor_Current^7^ - Motor’s current, which should present the following values: (i) close to 0 A when the compressor turns off; (ii) close to 4 A when the compressor is working offloaded; and (iii) close to 7 A when the compressor is operating under load (A);

### Digital sensors

The eight digital signals are collected directly from the APU and GPS information.

The digital sensors installed in the APU assume only two different values: zero when inactive or one when a specific event activates them. The considered digital sensors are the following.

- COMP - Electrical signal of the air intake valve on the compressor. It is active when there is no admission of air on the compressor, meaning that the compressor turns off or working offloaded.
- DV_electric - Electrical signal that commands the compressor outlet valve. When it is active, it means that the compressor is working under load; when it is not active, it means that the compressor is off or offloaded.
- TOWERS - Signal that defines which tower is drying the air and which tower is draining the humidity removed from the air. When it is not active, it means that tower one is working; when it is active, it means that tower two is working.
- MPG - Is responsible for activating the intake valve to start the compressor under load when the pressure in the APU is below 8.2 bar. Consequently, it will activate the sensor COMP, which assumes the same behaviour as the MPG sensor.
- LPS - Signal activated when the pressure is lower than 7 bars.
- Pressure_switch - Signal activated when pressure is detected on the pilot control valve.
- Oil_Level - The oil level on the compressor is active (equal to one) when the oil is below the expected values.
- Caudal_impulses - Signal produced by the flowmeter indicating the existence of the flow of air per second.

Regarding the GPS Information, the train was equipped with a secondary GPS antenna to collect the following:

- gpsLong - Longitude position (°).
- gpsLat - Latitude position (°).
- gpsSpeed - Speed (km/h).
- gpsQuality - Signal quality.

When the train is inside a tunnel and loses the satellite information, the acquisition system sets the GPS signal to 0.

## Data Records

The MetroPT dataset (available at Zenodo^8^) is included in a single file and reports data collected from the APU of an operating train from January to June 2022, which performs, on average, 26 trips per day. With a data acquisition rate of 1 Hz, the dataset is composed of 10979547 data points described by the above-referred 20 variables derived from analog and digital sensors installed in the train’s APU and its GPS coordinates, with no missing values (no pre-processing technique was applied on the dataset).

**Fig. 2:**
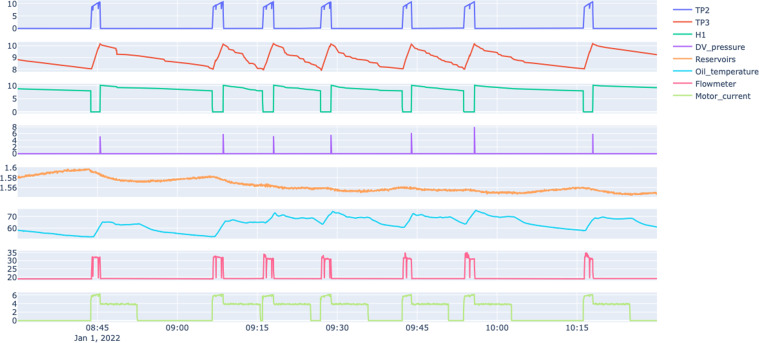
Snapshot of the analog sensors under normal operating conditions.

**Fig. 3:**
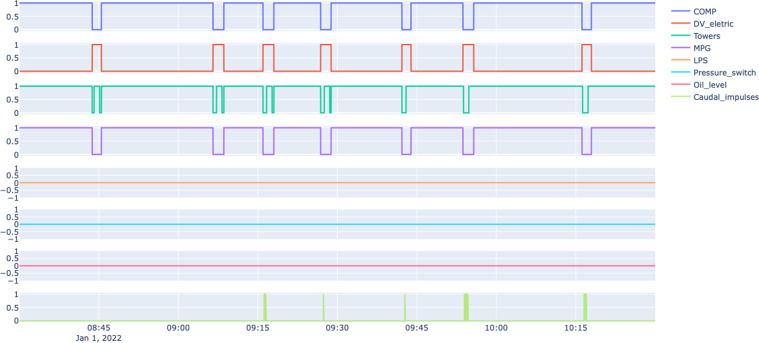
Snapshot of the digital sensors on the APU under normal operating conditions.

Figure 2 depicts a snapshot of the data collected by the eight analog sensors on a normal operating day (Jan 1, 2022) from 8:00 to 10:30. Figure 3 depicts a snapshot of the data collected by the digital sensors referring to the APU for the same period reported in Fig. 2, i.e. on a normal operating day (Jan 1, 2022) from 8:00 to 10:30.

**Fig. 4:**
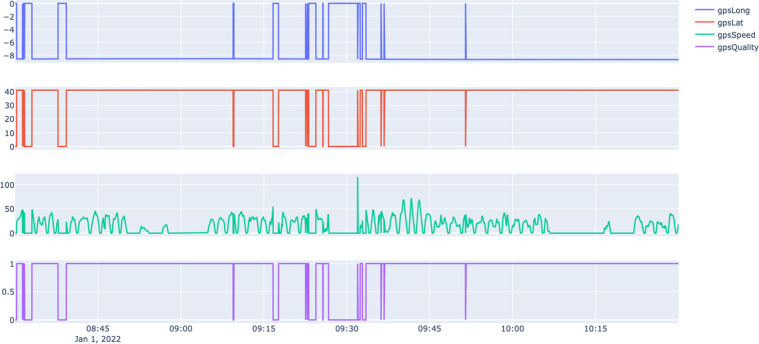
Snapshot of the GPS information under normal operating conditions.

**Fig. 5:**
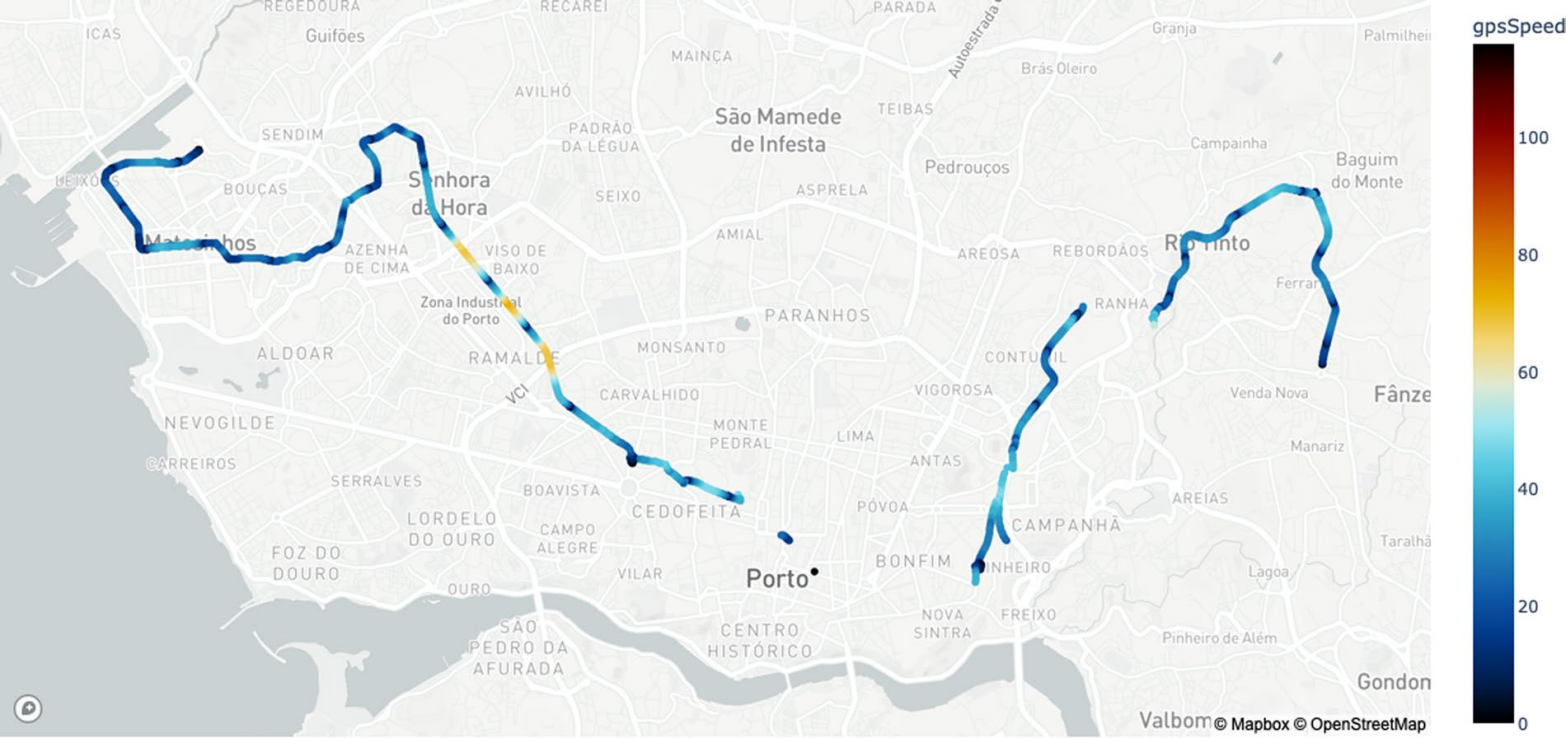
The GPS information on one of the train’s trajectories. When the train enters a tunnel loses the GPS signal.

In Fig. 4, we show the data collected from the GPS module, which includes latitude, longitude, speed and GPS signal quality, again for the same period reported in Fig. 2. The positional data is important to derive if the train is parked or in operation (cf. Table 1). The parking zones are typically located at the end of each line or in some underground parks. There are no missing values in this data. When the satellite information is lost by entering a tunnel (cf. Figure 5), it is set to 0.

**Table 1:** Latitude and longitude of the polygon points that define the location of the maintenance workshop and each parking.

Place	Polygon							
	Point 1		Point 2		Point 3		Point 4	
	Latitude	Longitude	Latitude	Longitude	Latitude	Longitude	Latitude	Longitude
Maintenance	41.212693	−8.661192	41.213258	−8.657555	41.215243	−8.660516	41.213375	−8.662785
Park 1	41.213604	−8.660474	41.212829	−8.661654	41.210198	−8.657824	41.211126	−8.656569
Park 2	41.376419	−8.757253	41.378367	−8.758960	41.378528	−8.758405	41.376558	−8.756909
Park 3	41.269800	−8.614621	41.272303	−8.613077	41.272428	−8.613600	41.271245	−8.614321
Park 4	41.168919	−8.543092	41.170735	−8.542713	41.170798	−8.543335	41.168919	−8.543646
Park 5	41.157216	−8.584129	41.156949	−8.583357	41.155669	−8.583958	41.155766	−8.584537
Park 6	41.113698	−8.607218	41.113636	−8.606551	41.115258	−8.606347	41.115251	−8.606870

## Technical Validation

### Reported failures

The ground truth was provided by the company using maintenance reports. According to the reported information, the dataset has three catastrophic failures (cf. Table 2) during six months. Two failures are related to air leaks in the system, and another is an oil leak. This technical information can be used to annotate the dataset.

**Table 2:** Failures disclosed on Maintenance Reports by type, failure component, start and end time and number of examples in the data set within that period.

Nr.	Type	Component	Start	End	# Exs.
Failure 1	Air Leak	Clients	28-02-22 21:53	01-03-22 02:00	14 820
Failure 2	Air Leak	Air Dryer	23-03-22 14:54	23-03-22 15:24	1 800
Failure 3	Oil Leak	Compressor	30-05-22 12:00	02-06-22 06:18	281 800

#### Failure 1 - Air leak on clients

This failure was an air leak on a pipe that feeds several clients on the systems, such as breaks, suspension, etc. The report provided by the maintenance teams showed a picture of a pipe that was blown up. In the second failure, the train recovered from the malfunction. In this case, the train needed to move to the maintenance building.

**Fig. 6:**
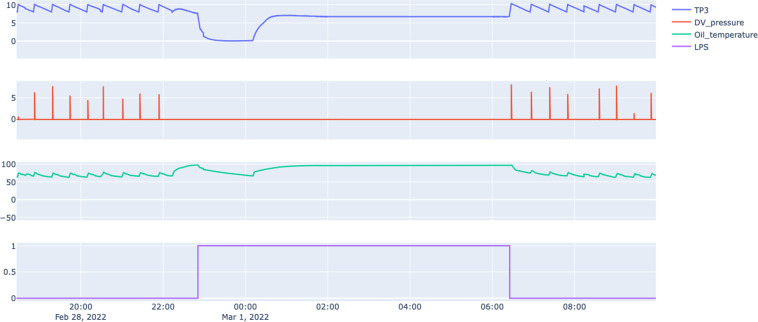
Snapshot of sensors information for Failure 1: Air Leak on Clients.

Figure 6 shows a catastrophic drop on the air pressure near 23:00 due to a broken air pipe. This problem was classified as a severe malfunction, and the train needed to be removed from operation.

#### Failure 2 - Air leak on air dryer

**Fig. 7:**
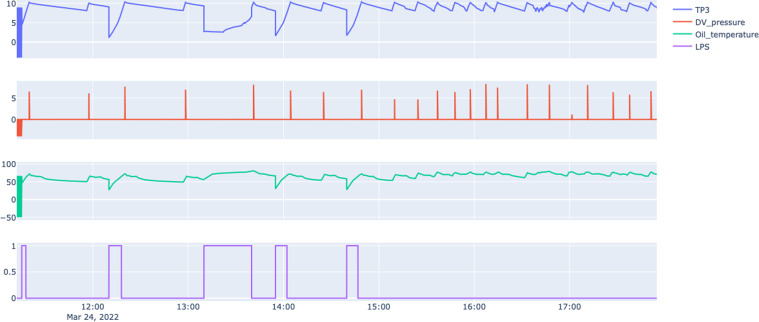
Snapshot of sensors information for Failure 2: Air Leak on Air Dryers.

The second failure is provoked by a malfunction of the pneumatic pilot valve that opens the drain pipes during the operation of the compressor. Figure 7 shows some anomalies on the regular fill of air tanks and consumption by the train clients. Between 12:00 and 14:00, we can observe huge drops in air pressure, provoking an alarm for the train driver (LPS warning variable), the compressor tries to compensate for the lack of air pressure, and the train continues in operation. After 15:00, the APU behaviour stabilises due to the return of the normal pattern of the pneumatic pilot valve.

#### Failure 3 - Oil leak on compressor

Regarding the oil leak, due to hardware design, there was not any signal system related to oil to warn the train driver. The oil leak provoked severe damage to the engine of the compressor, and subsequently, due to the inoperable compressor, it was observed a drop on the air pressure and the train needed to be removed from the tracks.

**Fig. 8:**
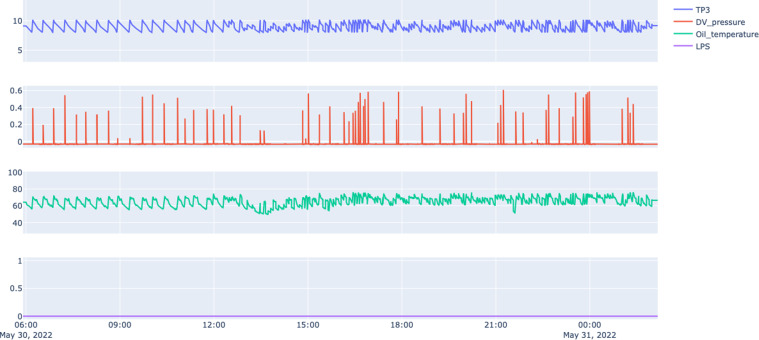
Snapshot of sensors information for Failure 3: Oil Leak on Compressor.

Figure 8 shows irregular patterns since 12:00 on the oil temperature, indicating that there is some issue with the oil system, we can also observe strange patterns on the air system, signalling that maybe the oil is escaping to the air system or the compressor is losing their efficiency.

### Evaluation protocol

The dataset can be used for two primary purposes:

1. predicting the occurrence of failures;
2. identifying the components involved in the failure.

**Fig. 9:**
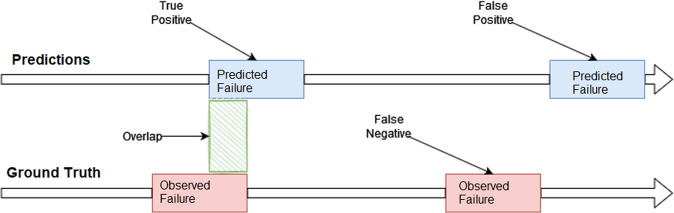
Proposed evaluation protocol for failure prediction (arrows indicate time).

For the first task predicting failures, the goal is to predict when it starts and the duration of the failure. The company established the need to detect the failure at least two hours before the train becomes non-operational to safely remove it from the tracks. In this scenario and for validation purposes, a failure is a time interval: start-end. The company also suggest the following evaluation protocol, also illustrated in Fig. 9:

- **True Positive (TP)** - when the predicted failure interval overlaps with the observed failure interval.
- **False Positive (FP)** - when the predicted failure interval does not overlap with the observed failure interval.
- **True Negative (TN)** - when there is no predicted failure, and there is no observed failure.
- **False Negative (FN)** - when there is no predicted failure, and there is an observed failure.

The overall goal is to minimize the number of false alarms (FP) and missed failures (FN), to avoid failures during the operational context and unnecessary maintenance actions.

For the first task of predicting failures, the objective is to discover the problems as early as possible after it manifests, i.e., to increase the overlap between the prediction and the ground truth. The second task is to identify the type of failure and in which component the failure occurs. Finally, it is crucial to compute the remaining useful life of the components to help the management team when they need to remove the train without provoking disruptions to the service.

Two recent works used the MetroPT dataset to propose methods for the failure prediction problem. In the first work^9^, the authors constructed a rule-based system to produce some alerts about the state of the compressor. The second work^10^ explores the usage of deep learning autoencoders to produce alerts. In both cases, the results are satisfactory, but there is a vast space to improve accuracy and explanation.

## Data Availability

The raw data collected and stored in a MySQL database was then converted to CSV formats using custom scripts in Python 3.8 with the library pandas for data manipulation. All plots supporting the fault descriptions were performed using Plotly library.
